# Provincial, Scotch, and Irish Hospitals


**Published:** 1893-10-21

**Authors:** 


### The Institutional Workshop.

### HOSPITAL ADMINISTRATION.

### PROVINCIAL, SCOTCH, AND IRISH HOSPITALS.*

In our previous articles we have confined our examination of the cost of administration to metropolitan hospitals general and special. These institutions, though differing widely from one another in certain respects, are almost identically situated so far as the giving public is concerned. When we come to consider the circumstances of Provincial, Scotch, and Irish Hospitals, considerable differences become apparent. A general hospital in the provinces is undoubtedly distinct from metropolitan hospitals of the same class, so far as the cost of administration is concerned. The general hospital of a provincial city is an object of great interest to all classes. The local papers continuously help the hospital by paragraphs and reports, and we are of opinion that metropolitan editors might, with advantage to themselves and their readers, as well as to the institutions, devote more space than at present to the work of our hospitals. Everything which happens in the history of a provincial hospital in the course of the year is duly made public. The provincial hospital very often occupies a commanding site, and few visitors of importance leave the town without being conducted over the hospital. For these and other reasons the adequate maintenance of a general hospital in the provinces becomes a part of the recognised duty of all classes, and every citizen who is worthy of the name considers it a privilege to help the work of the hospital, either with money or by personal service, or in some other way, according to his means and gifts. For these reasons, hospitals out of London do not advertise, and so a considerable saving in expenditure is effected. On the other hand, the number of wealthy givers in a provincial town are relatively few, compared with those who reside in the metropolis, at any rate for some portion of each year. Still the claims on the many are frequently pressing and numerous, whilst of those on the few, none have a better chance of liberal consideration than the hospital appeal.

In Scotland, where those who support the hospitals do so after due deliberation and for definite reasons, the collection of funds is relatively simple. We have known cases where the subscribers have positively looked forward to the call of the hospital collector, and it therefore becomes much easier to get in the subscriptions than in London. It follows that the cost of administration necessarily diminishes in the provinces because more than one large item of expenditure which the circumstances of a large city like London compel the hospital authorities to incur, is absent from the accounts of the provincial institutions. For this reason, the percentage of the cost of administration to that of maintenance, and the percentage of the cost of administration to total ordinary expenditure should be from 3 to 5 per cent. less in the provinces than it is in London.

Provincial Scotch and Irish General Hospitals.

| Name of Hospital. | Beds for Occupation. | Average Number Occupied. | Total In-patients Treated. | Total Out-patients Admitted. | Percentage of cost of Administration to that of Maintenance. | Percentage of Administration to Total Ordinary Expenditure. |
|---|---|---|---|---|---|---|
| *Provincial General Hospitals—* | | | | | | |
| Birmingham General Hospital ... ... | 271 | 233 | 4,612 | 53,566 | 10·460 | 9·422 |
| Bristol Royal Infirmary ... | 270 | 205 | 3,017 | 29,503 | 5·859 | 5·543 |
| Leeds General Infirmary ... | 436 | 315 | 5,778 | 37,704 | 9·971 | 9·067 |
| Liverpool Royal Infirmary | 295 | 241 | 3,384 | 10,519 | 6·112 | 5·759 |
| Manchester Royal Infirmary | 298 | 260 | 4,490 | 34,175 | 6·723 | 6·300 |
| Newcastle Royal Infirmary | 270 | 251 | 3,293 | 21,778 | 8·107 | 7·498 |
| Sheffield General Infirmary | 200 | 175 | 1,637 | 18,019 | 5·542 | 5·252 |
| *Scotch General Hospitals—* | | | | | | |
| Edinburgh Royal Infirmary | 710 | 652 | 8,637 | 23,000 | 8·533 | 7·862 |
| Glasgow Royal Infirmary ... | 582 | 560 | 5,800 | 20,526 | 6·061 | 5·715 |
| Glasgow Western Infirmary | 400 | 368 | 3,897 | 12,442 | 6·592 | 6·274 |
| *Irish General Hospital—* | | | | | | |
| Belfast Royal Infirmary ... | 189 | 141 | 2,455 | 20,293 | 8·223 | 7·599 |

Metropolitan and Provincial Hospital Expenditure Compared.

We have shown that 15 per cent. is a reasonable and fair maximum of the cost of administration to that of maintenance at the London hospitals, so that from 10 to 12 per cent. is the maximum sum for a provincial hospital to expend upon management. With a view to deciding the relative cost of administration to that of maintenance in all the principal hospitals in the country, we now give a table containing the names of general hospitals which, with one exception, have 200 beds and upwards, situated in the provinces, in Scotland, and in Ireland. The figures have been worked out from the reports, and are tabulated above. They relate as far as possible to institutions where the system of administration is practically the same as at the larger hospitals in London. Most of the Irish hospitals are much smaller than those in England, and we have, therefore, only included the Belfast Royal Infirmary in this table.

* Previous articles on this subject have appeared in The Hospital, page 333 for August 9th; page 349 for August 26th; page 381 for September 9th; page 413 for September 23rd; page 429 for September 30th; page 13 for October 7th; and page 29 for October 14th.

It will be seen that the higher maximum of 12 per cent. is not reached by any institution included in the table. At the Birmingham General Hospital the percentage of cost of administration to that of maintenance is practically 10½ per cent. (10·460), or 1½ per cent. below the maximum which we think should prove ample in the case of a provincial general hospital with an annual expenditure of £15,000. In every other case, the percentage of cost of administration to that of maintenance is less than 10 per cent., but at the Leeds General Infirmary it amounts to 9·971. In every case too the percentage of cost of administration to total ordinary expenditure is below 10 per cent., which is matter for congratulation.

Comparing the cost of management of metropolitan and provincial hospitals in 1892, we find that at the Royal Infirmary, Edinburgh, with 710 beds, of which 652 are constantly occupied, the cost of administration to that of maintenance was 8·533, whereas at the London Hospital, with 776 beds, of which 630 are constantly occupied, the cost of administration was less than 5½ per cent. Now these two institutions are fairly comparable in every respect, and if there be any advantage on either side, so far as the expenditure is concerned, it should be in favour of the Royal Edinburgh Infirmary, for the reasons we have already stated. Both the London Hospital and the Edinburgh Royal Infirmary have attained a deservedly high reputation for efficiency, and it is remarkable and satisfactory that the Committee of the London Hospital should spend three per cent. per annum less upon management than the managers of the Edinburgh Royal Infirmary. Such a fact as this should shame to perpetual silence those who have had the effrontery, without producing one tittle of evidence, to assert that the London Hospital is extravagantly administered.

Again, Guy's Hospital, with 600 beds, of which 433 are constantly occupied, cost 8·924 per cent. to administer; whilst the Glasgow Royal Infirmary, with 582 beds, of which 560 were constantly occupied, only expended 5¾ per cent. upon administration. We hold that these figures go to prove the costly unwisdom of keeping a large number of available beds empty throughout the year. It is fair to add that Guy's has only just adopted the uniform system of accounts, so that a much better result may be anticipated next year. At St. George's Hospital, with 355 beds, of which 225 were constantly occupied, the percentage of cost of administration to that of maintenance was only 5·339, compared with a cost of 6·592 per cent. at the Glasgow Western Infirmary, containing 400 beds, of which 368 were constantly occupied. Middlesex Hospital, with 320 beds, of which 254 were constantly occupied, spent 6·370 per cent. upon administration, as compared with the Manchester Royal Infirmary, containing 298 beds, of which 260 were constantly occupied, which spent 6·723 per cent. upon administration to that of maintenance. Other comparisons might be drawn, but we will refer our readers to The Hospital for August 19th, 1893, where the Metropolitan General Hospitals' figures will be found on page 334 for fear of wearying them by too many comparisons. It is not easy to explain why St. Mary's Hospital, with its 281 beds, of which 246 were occupied, should only cost 6·513 per cent. to administer, whilst the Birmingham General Hospital, with 271 beds, of which 233 were occupied, expended nearly 10½ per cent. upon administration compared with maintenance. These and other anomalies will become apparent if the figures in the two tables are closely studied, as we hope they will be. On the whole, most people will arrive at the conclusion that efficient and economical management is not the monopoly of either London, the Provinces, Scotland, or Ireland. All have good points, whilst some present openings for inquiry, if not for criticism.

These comparative tables prove that we are much nearer an accurate basis of comparison than ever before. The general adoption of the uniform system of accounts, the principle of which is now generally accepted and understood, has had much to do with this great change for the better in the cost of administration of our voluntary hospitals. We are glad also to know that the recent controversy on the London Hospital in the *Pall Mall Gazette*, and the action taken by The Hospital therein, has done much to increase public confidence in the voluntary system of hospital administration. We hope this increased confidence on the part of the public will speedily result in a much more liberal support too, as money was never more urgently needed by all classes of our hospitals than it is at the present time.

### Higher Hospital Officials and Pensions.

Further, and we particularly ask the editors of the metropolitan press to note this fact, the expenditure at the London includes the cost of the pension of £750 which the governors unanimously granted to Mr. W. J. Nixon, the late house governor. It is to the advantage of the voluntary hospitals and their supporters that the principal officials should be both specially trained and experienced, and that their rate of remuneration should be such as to justify the devotion of a life to the service of the hospital. The remuneration can never be magnificent, but it should be substantial enough to warrant men and women of the highest culture, training, and powers, to devote their lives to the conduct of the affairs of the great hospitals of this country. Five per cent. of the income would not be an extravagant sum to pay in salaries to the chief officials, and we are convinced that as a matter of business, hospital committees would be wise to look into this question of remuneration, and to raise the rate of secretary and superintendents. At the larger hospitals, say from £400 to £800 or £1,000 per annum, according to their size and importance. The salary of the lady superintendent, too, of this type of hospital, might be raised from £150 to £250 per annum, provided such salaries collectively do not amount to more than 5 per cent. of the annual expenditure. A most cruel and wanton attack was made upon Mr. Nixon because the governors granted him a pension upon which he could live in comfort in his old age, after 40 years' magnificent service. The fact that the London Hospital is managed at a cost of 3 per cent. less than the Edinburgh Royal Infirmary, justifies the payment of any reasonable pension to the higher officials of the former institution. A pension is essential, in our opinion, to the well-being of the institutions, because it enables an officer or a servant to devote his or her whole energies and strength to the work, apart from any personal considerations whatever. The Royal National Pension Fund, by its system of federation for institutions and committees, enables every hospital and kindred institution to contract itself out of all liability for pensions at an expenditure of about one-third of the cost of the annual value of the annuity which will ultimately be received. There is, therefore, no excuse for any committee who wish to do their work thoroughly if they do not at once adopt a pension scheme adequate to the requirements of their staff. Many of the best managed hospitals have already become affiliated to the Royal National Pension Fund, 8, King Street, Cheapside, E.C., and it would be to the public interest if the majority were to follow their wise example.

